# Epidemiological Study and Molecular Characterization of Lumpy Skin Disease in Cattle in Egypt

**DOI:** 10.1155/vmi/2835566

**Published:** 2026-02-27

**Authors:** Heba Hassan El-Nady, Ahmed Mansour, Mohamed Ibrahim Eissa, Naser Zeidan Abou-Zeid, Elshaima Mohamed Fawzi, Amal Mokhtar Abd El-Raof, Abdelrhman Awad Sobeih, Mohamed Mansour Bakrash, Yousry Abdelfatah El Shazly

**Affiliations:** ^1^ Department of Animal Medicine, Infectious Diseases, Faculty of Veterinary Medicine, Zagazig University, Zagazig, 44511, Egypt, zu.edu.eg; ^2^ Department of Pathology, Faculty of Veterinary Medicine, Zagazig University, Zagazig, 44511, Egypt, zu.edu.eg; ^3^ Virology Department, Tissue Culture Unit, Animal Health Research Institute, El-Giza, 12311, Egypt, ahri.gov.eg; ^4^ Animal Wealth Development Department, Faculty of Veterinary Medicine, Zagazig University, Zagazig, 44511, Egypt, zu.edu.eg; ^5^ Private Sector, Beheira, Egypt; ^6^ Faculty of Veterinary Medicine, Veterinary Hospital, Zagazig University, Zagazig, 44511, Egypt, zu.edu.eg

## Abstract

Lumpy skin disease virus (LSDV) constitutes one of the most significant poxvirus infections impacting livestock and has a high morbidity rate and a comparatively low mortality rate. This study was designed to elucidate the epidemiology and the molecular analysis of LSDV using conventional polymerase chain reaction (PCR) to amplify the extracellular enveloped viral (EEV) glycoprotein gene. Out of 470, 167 cows from 10 herds exhibited typical signs of LSD in four governorates in Egypt (Al‐Sharkia, Al‐Ismailia, Al‐Menofia, and Al‐Beheira) during recent outbreak in summer 2025. The morbidity, mortality rate, and case fatality rates were 35.53% (167/470), 5.32% (25/470), and 14.97% (25/167), respectively. The univariable logistic regression result demonstrated that age of the animal, grazing system, water source, and introduction of new animals without quarantine were significant predictors for the outbreak of LSD. Vaccination of the animals with using fly repellent was recommended to control the disease. Sixty‐nine out of 82 (84.1%) developed pock lesions on chorioallantoic membrane, while 75 out of 82 (91.46%) had cytopathic effects on Madin‐Darby Bovine Kidney cell line. Twenty‐two out of 23 (95.65%) samples tested positive for PCR at 958 base pair. The partial sequence of 3 samples and ARRIAH LSD VAC was translated into amino acids revealing a distinct 27‐nucleotide insertion with substitution of 11 nucleotides when compared to ARRIAH LSD VAC; consequently, there was a variation in more than 10 amino acids. The field isolates presented single‐nucleotide polymorphism (SNP) at position 54 G (ZAG‐LSD3)/A (ISM‐LSD1 and MNF‐LSD2), exhibiting a high degree of nucleotide similarity with a virulent strain from Egypt, India, and Austria. Furthermore, the partial sequence of the EEV glycoprotein gene possesses the capacity to implement the differentiation between infected and vaccinated animals (DIVA) strategy when utilized alongside the ARRIAH LSD VAC, which has recently been employed in Egypt.

## 1. Introduction

Lumpy skin disease (LSD) is a devastating transboundary skin disease affecting cattle and is caused by the lumpy skin disease virus (LSDV). LSDV, goatpox virus (GTPV), and sheeppox virus (SPPV) are members of the Poxviridae family of the genus Capripoxvirus (CaPVs). CaPVs are double‐stranded DNA molecule [1]. It is approximately 150 kbp long and is thought to contain 156 genes, with a coding density of 95%. It is comprised of a primary coding region and two identical inverted terminal repeats (ITR). Six identified LSDV proteins are believed to play a role in suppressing or modifying the host’s immune response [2].

LSD is an endemic disease in Africa, with the first known incidence occurring in Zambia in 1929. In Egypt, LSDV was first identified in the summer of 1988 in the Suez Canal governorate, with a recurrence in 2006 after importing livestock cattle from Ethiopia [3]. In spite of expansive vaccination programs, the disease spread quickly in various regions in Sharkia Governorate in three successive years (2018, 2019, and 2020) [4], across three Egyptian governorates (Dakahlia, El‐Menia, and El‐Fayoum) [5], and in Beni‐Suef Governorate [6].

LSD is primarily spread by arthropod vectors like hard ticks, mosquitoes, and flies as well as via the saliva, nasal secretions, and semen of infected animals [7]. The disease causes low mortality of 1%–10% and significant morbidity of up to 85% [8], and all ages and cow breeds are at high risk of susceptibility. LSD is economically significant due to the direct and indirect losses associated with a sharp drop in milk production, weight loss, infertility, lower‐quality hides, chronic impairment, abortion, and mortality [9].

The typically clinical symptoms of LSD diseased animals are fever, skin nodules, emaciation, enlarged lymph nodes, leg edema, and death in complicated cases [10]. The host’s immunological condition, the vector’s distribution, and the virulence of the strains are the primary factors influencing the severity and epidemiology of LSD [11].

Since only symptomatic therapy is advised to prevent LSDV infection [12], the primary control methods are vaccination in conjunction with vector management and controlled quarantine procedures [13]. Cattle in Egypt vaccinated with the Romanian SPPV vaccine received warnings concerning the partial cross‐protection [14]. Moreover, live attenuated Neethling strain LSDV vaccines were used in Egypt [15], recently introduced in 2021 [16]. After a successful mass vaccination campaign, the ability to distinguish between infected and vaccinated animals (DIVA) is necessary to achieve disease‐free status [17]. Various methods leveraging PCR, real‐time PCR, and HRM techniques have been established for identifying the LSDV genome. Molecular epidemiological studies of LSDV focus on analyzing genomic segments such as the GPCR, RPO30, P32, and extracellular enveloped viral (EEV) glycoprotein genes [18]. To distinguish between LSDV vaccination strains and the wild‐field type of LSDV generated from diseased cow specimens, the LSDV putative EEV glycoprotein target genome was thus explicitly selected [19]. EEV is a protein‐based outer envelope found on LSDV that enable virus to multiply within the host, aid in immune evasion by obscuring viral antigens, and adhering to the outer LSDV envelope. Also, this gene might be essential for developing effective LSD vaccinations and/or diagnostic techniques [20]. An analysis of the genetic sequences of LSDV based on EEV glycoprotein between vaccine strains and wild field strains revealed that the vaccinal strains lacked 27‐nucleotide that was specific to the wild‐field strain type of LSDV [18].

This study aimed to analyze the epidemiological conditions and risk factors associated with LSDV during recent outbreak at 2025 by isolating LSDV from suspicious cows using traditional techniques and confirming identification through PCR targeting the EEV glycoprotein gene. The analysis focused on the genetic profile of circulating LSDV in four Egyptian governorates and explored the genetic link between these strains and other LSDV strains, including vaccine strains. Additionally, this study was carried out to ascertain whether the observed clinical cases was attributable to a field strain or a vaccinal strain through the DIVA approach, which was based on a partial sequence of the EEV glycoprotein gene of the representative field isolates and ARRIAH LSD VAC.

## 2. Materials and Methods

### 2.1. Animals

During the recent LSD outbreak in summer 2025 in four northern Egyptian governorates: Al‐Sharkia, Al‐Ismailia, Al‐Menofia, and Al‐Beheira. One hundred and sixty‐seven out of 470 cows from 10 herds were suspected of LSD infection upon clinical examination according to Constable et al. [21]. Most animals were raised on flock grazing pattern with communal water source and others reared on separately, and the data about animals and herds were recorded as illustrated in Table 1.

**TABLE 1:** Epidemiological characteristics of LSD outbreak.

Factors	Subfactor	Total no. of animals	No. of diseased animals	Morbidity rate (%)
Age	< 1 Year	47	5	10.64
	1–3 Year	273	115	42.12
	> 3 Year	150	47	31.33

Sex	Female	315	119	37.78
	Male	155	48	30.97

Grazing pattern	Flock	263	107	40.68
	Individual	154	56	36.36
	Mixed	53	4	7.55

Governorate	Al‐Sharkia	164	50	30.49
	Al‐Ismailia	200	85	42.50
	Al‐Menofia	36	12	33.33
	Al‐Beheira	70	20	28.57
	Total	470	167	35.53

Breed	Baladi	145	48	33.10
	Mixed	100	40	40.00
	Holestin	225	79	35.11

Communal water source	Yes	220	117	53.18
	No	250	50	20.00

Introduction of new cattle	Yes	290	120	41.38
	No	180	47	26.11

Fly replant	Yes	155	35	22.58
	No	315	132	41.90

Vaccination	Yes	180	32	17.78
	No	126	53	42.06
	UnKnown	164	82	50.00

Free animal movement	Yes	295	113	38.31
	No	175	54	30.86

Contact with other animals	Yes	230	80	34.78
	No	240	87	36.25

### 2.2. Samples

Eighty‐two samples (*n* = 75 skin nodules and *n* = 7 nasal swabs) were collected from diseased cows. Skin nodules were collected under sterile surgical techniques to remove all nodules as previously described by Bihonegn and Feyisa [22]. Nasal swabs and skin nodule samples were ground in a sterile mortar with sterile sand, and the suspension supernatant was collected in a sterile Eppendorf tube, and an equal volume of sterile phosphate buffered saline (PBS) containing streptomycin sulfate (1 mg/mL), sodium penicillin (1000 IU/mL), and fungizone (amphotericin 2.5 μg/mL) was added to prevent bacterial and fungal contamination [23]. The tubes were then transported in a cold box to the Virology Department of the Animal Health Research Institute in Dokki, El‐Giza. The samples were kept for later processing at −80°C.

### 2.3. Laboratory Diagnosis

#### 2.3.1. Isolation of Suspected LSDV Samples on Embryonated Chicken Eggs (ECEs) and Cell Culture

Two hundred μL of each sample’s supernatant fluid were inoculated into the chorioallantoic membrane (CAM) of specific pathogen free (SPF) of ECEs aged 13 days. The virus was isolated through the CAM route in a 13‐day old incubated for 5 days at 37°C, and the infected CAMs were examined daily for characteristic pock lesions [24]. Furthermore, 300 μL of the supernatant fluid was cultured into a flask containing Madin‐Darby Bovine Kidney (MDBK) cell culture propagated in Dulbecco’s Modified Eagle’s Medium (DMEM) and supplemented with 10% fetal calf serum and antibiotics. The flasks were examined daily under a microscope to detect signs of CPE development, and virus isolates from the 3rd passage were labeled and kept at −80°C for molecular diagnosis [23]. The MDBK (NBL‐1) cell line was supplied from the Virology Department of the Animal Health Research Institute and originated from the kidney of male bovine. It was mycoplasma‐ and BVDV‐free, and it was authenticated for the described experiment (RRID: CVCL_0421).

#### 2.3.2. Molecular Diagnosis of LSDV

For the molecular diagnosis of field samples, twenty‐one samples were taken from clinically diseased cows (16 skin biopsies and 5 nasal swabs). Extraction of viral DNA was applied according to the instructions for the QIAamp DNA minimal kit (source: Metabion, Germany), which was used to acquire viral particles. The EEV glycoprotein gene primer forward 5′‐ATG​GGA​ATA​GTA​TCT​GTT​GTA​TAC​G‐3′ and reverse 5′‐CGA​ACC​CCT​ATT​TAC​TTG​AGA​A‐3′ were provided by Metabion (Germany) [25]. The reaction procedures were denaturation at 94°C for 5 min, 35 cycles at 94°C for 30 s, 55°C for 40 s, and 72°C for 1 min, followed by a final extension of 72°C for 7 min.

A transilluminator was used to view the PCR products following their electrophoresis in a 1.5% agarose gel [26].

In the purification protocol, the QIAquick PCR Product extraction kit (Qiagen Inc., Valencia, CA) is used. A purified PCR product of three field samples (skin nodules) was collected from 3 cows (1st cow during June 2025 after vaccination with the AL‐Abassya LSD vaccine, 2nd cow during September 2025 after vaccination with an unknown type of LSD vaccine, and 3rd cow during September 2025; it is not known if it was vaccinated or not) and the Neethling strain of ARRIAH LSD VAC (The trade name for ARRIAH LSD vaccine, and it is commercial Russian vaccine, was recently introduced in Egypt in 2025 and manufactured by the Federal Center for Animal Health (FGBI″ARRIAH″)), was sequenced in one direction on an Applied Biosystems 3130 automated DNA sequencer (ABI, 3130, USA), utilizing a BigDye Terminator V3.1 cycle sequencing kit (Cat. No. 4336817) with a ready reaction (Perkin‐Elmer/Applied Biosystems, Foster City, CA).

The CLUSTAL W multiple sequence alignment approach, created by Thompson et al. [27] and accessible in Version 1.83 of the MegAlign module of Lasergene DNAStar software Pairwise, was used to compare the sequences. MEGA6 was used to conduct phylogenetic analyses utilizing maximum likelihood, neighbor joining, and maximum parsimony [28].

### 2.4. Statistical Analysis

Univariable logistic regression (ULR) was performed to assess the relationship between potential risk factors and the prevalence of LSD in cattle. To assess the robustness of univariable associations and explore potential geographic confounding, disease occurrence was examined across governorates by stratified analyses. The results are shown as odds ratios (OR) with 95% confidence intervals (CI); statistical significance was indicated by a value of *p* < 0.05. Statistical Package for Social Sciences (SPSS, IBM Corp., Armonk, NY) Version 24.0 was used for all analysis and charts.

## 3. Results

### 3.1. Clinical Finding on Field Investigation

**FIGURE 1:**
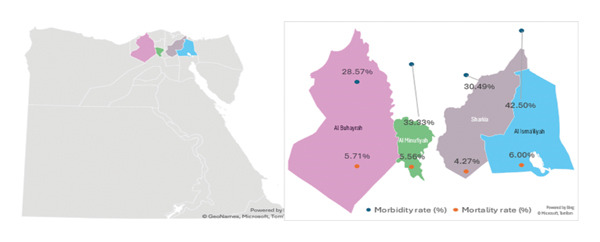
A map of Egypt displaying the investigated governorates with the morbidity and mortality of LSDV.

**FIGURE 2:**
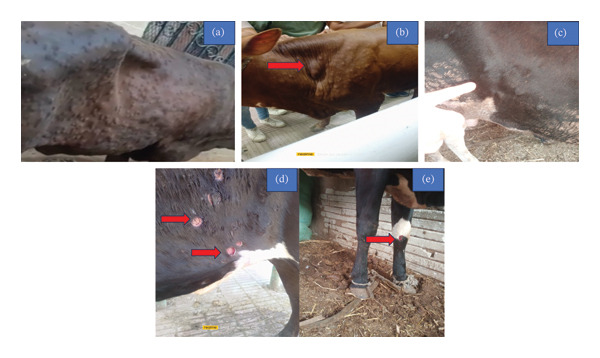
Diseased animals show typical signs of LSDV. (a) The active nodular skin lesions that are widespread around the entire body, (b, c) enlargement of prescapular and prefemoral L.N, and (d, e) sitfast on abdomen and leg with edema in leg.

Out of 470 cows from 10 herds of cows in four governorates (Al‐Sharkia, Al‐Ismailia, Al‐Menofia, and Al‐Beheira) during recent outbreak summer 2025, about 167 showed the usual clinical symptoms of LSD with the morbidity rate 35.53% (167/470); the epidemiological data recorded a 5.32% (25/470) and 14.97% (25/167) mortality rate and case fatality rate, respectively, as mentioned in Table 2 and Figure 1. Multiple cutaneous nodules of various sizes appeared suddenly throughout the body of the clinically infected cows. They also had pyrexia, excessive salivation, appetite loss, nasal and ocular discharge, and enlargement of the superficial lymph nodes, particularly the prefemoral and prescapular. During the initial 2‐3 days after pyrexia (39.5°C–41°C), the cutaneous nodules presented as small elevated lesions, which subsequently evolved into nodules gradually increasing to 2‐3 cm in diameter within a span of 7–10 days. Thereafter, approximately 2 weeks postappearance, these nodules underwent rupture, necrosis, and ulceration with a slender margin of hemorrhage that is marked by the distinctive pathology, known as the “inverted cone‐shaped area” of necrosis or “sit fast,” is discernible along the thin bleeding border (Figure 2(a), 2(b), 2(c), 2(d), and 2(e)). There are some cases associated with an unfavorable prognosis, including pneumonia (45/167) 26.9%, edema in the brisket and extremities (21/167) 12.57%, S/C abscess (8/167) 4.8%, and bloody urine (12/167) 7.1%.

**TABLE 2:** Morbidity, mortality, and case fatality rates of LSD in 4 governorates.

Governorate	Total no. of animals	No. of diseased	Morbidity rate (%)	No. of death	Mortality rate (%)	Case fatality (%)
Al‐Sharkia	164	50	30.49	7	4.27	14.00
Al‐Ismailia	200	85	42.50	12	6.00	14.12
Al‐Menofia	36	12	33.33	2	5.56	16.67
Al‐Beheira	70	20	28.57	4	5.71	20.00
Total	470	167	35.53	25	5.32	14.97

### 3.2. ULR

ULR was used as an exploratory tool to screen for potential associations between selected factors and LSD occurrence (Table 3). The predictors included in the univariable analysis were selected based on known or suspected epidemiological relevance to LSD transmission and management practices; however, these findings should be interpreted cautiously as they do not account for confounding. The age of the animal was a strong and significant predictor for LSD. Animals younger than 1 year had substantially lower odds of disease compared with animals aged 1–3 years (OR = 0.16, *p* < 0.001), while animals older than 3 years also showed reduced odds (OR = 0.63, *p* = 0.02). Grazing pattern showed variable associations. Mixed grazing systems were associated with markedly lower odds of LSD (OR = 0.14, *p* < 0.001), whereas flock grazing was not significantly different from individual grazing (OR = 1.20, *p* = 0.38). Locality was significantly associated with LSD occurrence. Compared with Al‐Ismailia, cattle in Al‐Sharkia (OR = 0.59, *p* = 0.019) and Al‐Beheira (OR = 0.54, *p* = 0.041) had lower odds of the disease. Management‐related variables, including avoidance of communal water sources, monitoring of new animal introduction, and use of fly repellents, were associated with reduced odds of LSD. Vaccination status was also significantly associated with LSD occurrence; vaccinated animals showed lower odds compared with animals of unknown vaccination status (OR = 0.22, *p* < 0.001). However, these associations represent unadjusted effects and should be considered hypothesis‐generating rather than confirmatory. Stratified analyses by governorate demonstrated a consistent direction of association with the univariable regression results (Table 4), supporting the robustness of observed exploratory trends. Al‐Ismailia consistently exhibited the highest morbidity and mortality rates, whereas Al‐Sharkia and Al‐Beheira showed lower disease burden. These exploratory findings warrant further investigation using multivariable logistic regression to identify independent predictors.

**TABLE 3:** Univariable logistic regression model of risk factors associated with LSD occurrence in cattle.

Factors	Subfactor	No. of diseased (%)	Exp (B)‐OR	95% C. I	*p* value
Age	1–3 Year (REF)	115 (42.12)			
	< 1 Year	5 (10.63)	0.16	0.06–0.43	<0.001^S^
	> 3 Year	47 (31.33)	0.63	0.41–0.95	0.029^S^

Sex	Female (REF)	119 (37.78)			
	Male	48 (30.97)	0.74	0.49–1.11	0.148^NS^

Grazing pattern	Individual (REF)	56 (36.36)			
	Flock	107 (40.68)	1.20	0.80–1.81	0.383^NS^
	Mixed	4 (7.55)	0.14	0.05–0.42	<0.001^S^

Governorate	Al‐Ismailia (REF)	85 (42.5)			
	Al‐Sharkia	50 (30.49)	0.59	0.38–0.92	0.019^S^
	Al‐Menofia	12 (33.33)	0.68	0.32–1.43	0.305^NS^
	Al‐Beheira	20 (28.57)	0.54	0.30–0.98	0.041^S^

Breed	Holistien (REF)	79 (35.11)	0.92	0.59–1.42	
	Baladi	48 (33.10)	1.23	0.76–2.00	0.691^NS^
	Mixed	40 (40)			0.399^NS^

Communal water source	Yes (REF)	117 (53.18)			
	No	50 (20)	0.22	0.15–0.33	<0.001^S^

Introduction of new cattle	Yes (REF)	120 (41.38)			
	No	47 (26.11)	0.50	0.33–0.75	0.001^S^

Fly replant	No (REF)	132 (41.90)			
	Yes	35 (22.58)	0.40	0.26–0.63	<0.001^S^

Vaccination	Unknown (REF)	82 (50)			
	Yes	32 (17.78)	0.22	0.13–0.35	<0.001^S^
	No	53 (42.06)	0.73	0.45–1.16	0.180^NS^

Free animal movement	Yes (REF)	113 (38.31)			
	No	54 (30.86)	0.72	0.48–1.07	0.103^NS^

Contact with other animals	Yes (REF)	80 (34.78)			
	No	87 (36.25)	1.07	0.73–1.56	0.740^NS^

*Note:* REF = reference category; Significance determined at *p* < 0.05; S = significant.

Abbreviations: CI = confidence interval, NS = not significant, and OR = odds ratio.

**TABLE 4:** Stratified distribution of LSD outcomes by governorate.

Governorate	Morbidity (%)	Mortality (%)	Direction vs Al‐Ismailia
Al‐Ismailia	42.50	6.00	Reference
Al‐Sharkia	30.49	4.27	Lower
Al‐Menofia	33.33	5.56	Lower
Al‐Beheira	28.57	5.71	Lower

### 3.3. Isolation of Suspected LSDV Samples on ECEs and Cell Culture

**FIGURE 3:**
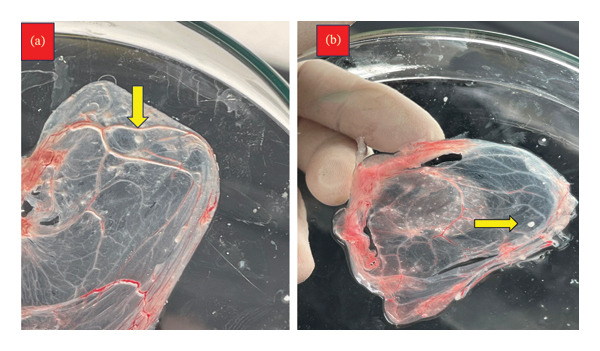
The characteristic pinhead pock lesions on CAM of SPF‐ECEs with congestion of blood vessels post 3 passages (as referred by yellow arrows).

**FIGURE 4:**
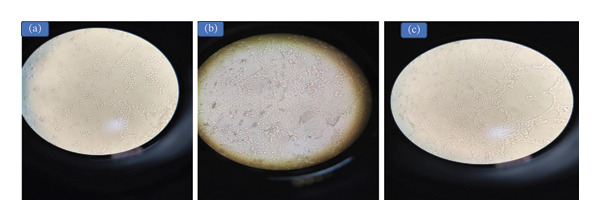
The characteristic CPE on MDBK cell line, (a, b) cells become rounding and exhibit aggregation in grape‐like shape and (c) cells reveal severe detachment and vacuolation 5 days postinoculation.

On ECEs, 69 out of 82 (84.1%), skin nodule biopsies 66/75 (88%), and nose swabs 3/7 (42.9%) that were infected in ECE developed characteristic pock lesions. The pock lesions begin to be noticeable after the 1st to the 3rd passages and appear as opaque, tiny white foci, round, pinpoint to pinhead in size, and dispersed across the inoculation site with a small thickening and edema of the membrane and congestion of blood vessels (Figure 3), there are not any signs of pock lesions in the other 13 samples. While on MDBK cell culture, 75 out of 82 (91.46%), skin nodule biopsies 71/75 (94.67%), and nasal swabs 4/7 (57%) displayed cytopathic effects (CPE) that were characteristic of LSDV, as evidenced by three consecutive blind passages. The observable traits of CPE include cellular rounding, aggregation of cells, and the coalescence of cells to form clusters. With the passage of time, the severity of CPE increased, ultimately leading to the detachment of most of the cellular monolayer. This finding was juxtaposed with the morphology of a standard, uninfected monolayer utilized as a negative control (Figure 4).

### 3.4. Molecular Identification of LSDV in Clinical Samples

**FIGURE 5:**
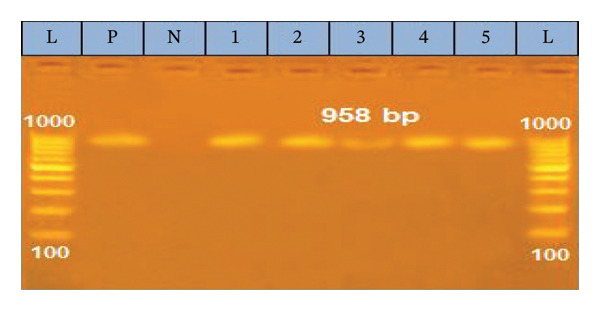
Agarose gel electrophoresis of PCR product showing the amplification of fragment of EEV glycoprotein gene at 958 bp. 1000 bp DNA ladder (L), positive control (P), negative control (N). Lane (1, 2, 3, 4, 5): exhibited positive amplicons for LSDV.

In the current study, we used conventional PCR to detect the DNA of LSDV, which relies on partial amplification of the EEV glycoprotein gene (958 bp). The results showed that 22 out of 23 (95.65%) samples tested positive for LSDV, {1 out of 2 nasal swab samples and all lsd21 skin nodule biopsies (9 samples had pock lesions, 9 samples causing CPE on MDBK, and 3 negative skin biopsy samples by culture on both ECE and MDBK)} (Figure 5).

### 3.5. Sequence and Phylogenetic Analysis of EEV Glycoprotein Gene

The partial EEV glycoprotein gene was amplified and sequenced from three representative samples and one commercial live attenuated vaccine (ARRIAH LSD VAC) available in Egypt, these registered in GenBank with accession numbers PX046541, PX046542, PX046543, and PX046546. The phylogenetic analysis of the EEV gene sequences revealed three clusters: LSDV, SPPV, and GTPV to identify the nearby areas of disease transmission responsible for recurrent outbreaks.

**FIGURE 6:**
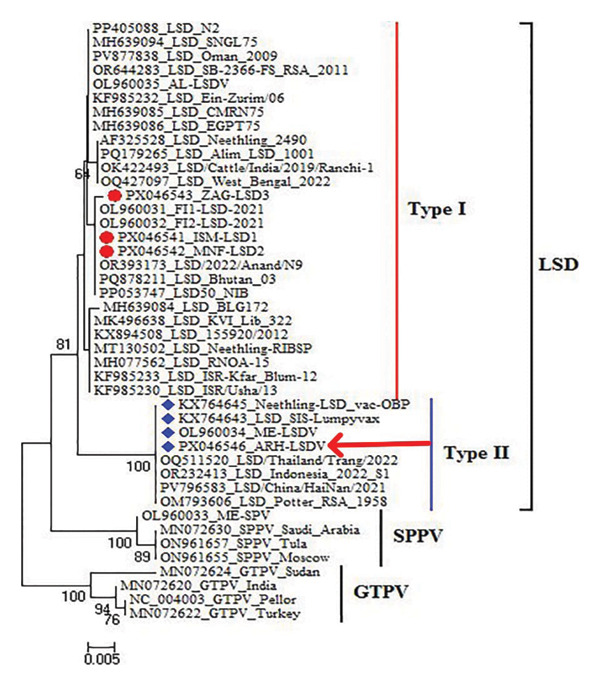
Phylogenetic analysis based on partial sequence of EEV glycoprotein gene of three field isolates marked with red circle and ARRIAH LSD VAC labeled by red arrow and other isolates published in GenBank.

**FIGURE 7:**
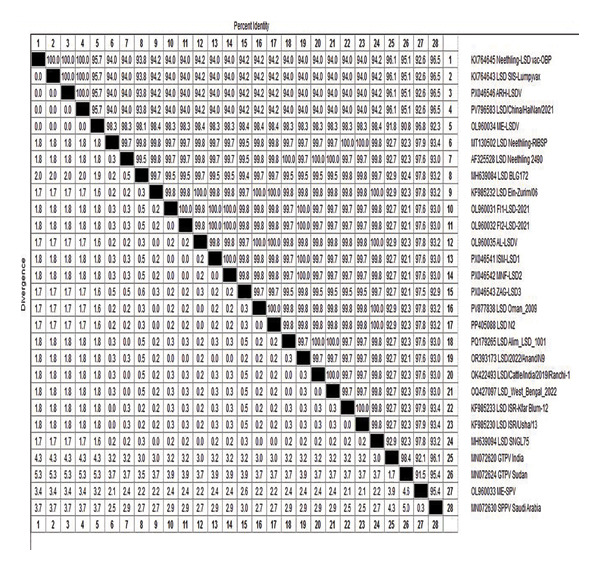
Pair wise sequence distance for EEV glycoprotein gene of 3 local LSDV isolates from different governorates and Neethling vaccine of ARRIAH LSD VAC with reference LSDV, sheep pox virus, and goat pox virus available in GenBank.

All LSDV strains in this investigation were categorized among LSDV clusters, where the investigated strain divided into two subclusters: field strains (Type I) and vaccine strains (Type II) (Figure 6). The nucleotide and deduced amino acid sequences of the EEV gene from 3 diseased cows and ARRIAH LSD VAC aligned with those retrieved from Gene Bank revealing that isolates from Al‐Ismailia and Al‐Menofia had 100% genetic identity compared to isolates from Al‐Sharkia (99.85%), and these field isolates exhibited 94.27%–94.42% identity with the ARRIAH LSD VAC. Overall, the LSDV isolates shared high nucleotide sequence similarity with a virulent Egyptian strain, ranging from 99.7% to 100%, as well as exhibited more closely genetic link with other LSDV sequences from various regions including Africa, Asia, Europe, and USA. Moreover, the LSDV isolates showed significant genetic similarity of 99.85%–100% with sequences from India 2024 (OR393173) and Austria 2023 (PQ878211). In Comparisons with other strains, it revealed 99.85%–99.7% identity with South Africa 2024 (OR644283), UK 2025 (PV877838), 99.7%–99.55% with USA 2025 (AF325528), and Bangladesh 2024 (PQ179265). In contrast, these isolates had lower similarity 94.27%–94.42% with Indonesia 2022 (OR232413), China 2021(PV796583) and Thailand 2022 (OQ511520). Additionally, field isolates shared a 99.85%–99.7% identity with AL‐LSDV (OL960035) and 98.34%–98.49% with ME‐LSDV (OL960034). However, those outbreak sequences exhibited low identities ranging from 94.27% to 94.42% when compared to other LSDV vaccination strains as LSD_SIS‐Lumpyvax_vaccine (KX764643). Also, significant sequence differences were recorded between field isolates and GTPV isolates (92.61% identity) and SPPV isolates (93.36%–93.21% identity), indicating substantial diversity with the current isolates (Figure 7).

**FIGURE 8:**
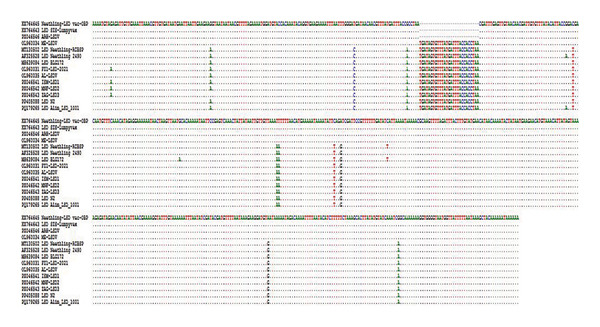
Multiple sequence alignments of the partial nucleotide sequences of the of EEV glycoprotein gene of 3 local LSDV isolates from different governorates in Egypt and Neethling vaccine of ARRIAH LSD VAC with representative sequences of LSDV retrieved from GenBank. A distinctive sequence signature of 27 nucleotides only in LSDV Neethling‐like viruses.

The multiple sequence alignment of the current field isolates revealed a single‐nucleotide polymorphism (SNP) at position 54 G (ZAG‐LSD3)/A (ISM‐LSD1 & MNF‐LSD2) across the isolates of outbreaks in Al‐Sharkia, Al‐Ismailia and Al‐Menofia. The genetic diversity among the three sequenced field isolates indicates a distinct 27‐nucleotide insertion and substitution of 11 nucleotides compared to ARRIAH LSD VAC. In contrast, variations between these field isolates and those retrieved from GenBank were limited to two and/or three SNPs. Moreover, the difference between these isolates and other LSD sequences related to Neethling vaccines was shown to be 11 nucleotide substitutes with deletion of 27 nucleotide (Figure 8).

**FIGURE 9:**
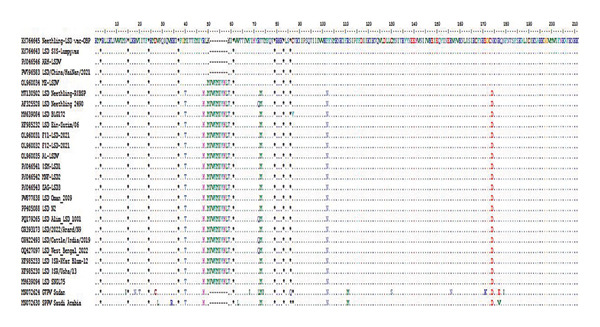
Amino acid sequences alignment of EEV glycoprotein gene of the three current isolates from different localities in Egypt and ARRIAH LSD VAC and other LSD either isolates or vaccinal strains and SPPV and GTPV retrieved from database of GenBank.

Furthermore, the 211 deduced amino acid sequences of the EEV glycoprotein gene from field isolates indicated a five‐amino‐acid variation at various sites: M/T at Position 40, R/N at Position 48, T/M at Position 73, F/N at Position 102, and N/D at Position 174. Whereas, vaccinal strains of LSD exhibited a deletion of 9 A. As with substitution of 5 more AAs compared to all both field isolates and GenBank isolates. However, in comparison with goatpox, 14 amino acids were substituted, while 5 AAs substitutions were recorded with sheeppox (Figure 9).

## 4. Discussion

LSD is a poxviral illness that affects cattle, particularly cattle of all ages, and has a significant socioeconomic impact on animal livestock [29].

According to OIE, the disease is a notifiable disease, and it is endemic in Egypt and other Middle Eastern nations, affecting cattle [30]. Thus, it is crucial to recognize and track viral infections quickly.

In summer 2025, LSD emerged in Egypt, hitting the four governorates (lL‐Sharkia, Al‐Ismailia, Al‐Menofia, and Al‐Beheira). Infected cows exhibited clinical symptoms that vary in severity among animals, including fever, respiratory distress, lacrimation, enlargement of superficial lymph nodes, and edema of the leg in certain animals. Moreover, the cow had scattered skin nodules that progressed to necrotic, ulcerating, and ultimately deep scabs, aligned with the results in [16, 31]. Furthermore, some seriously infected animals showed pneumonia and brisket edema as documented by Rouby et al. [32]. In this scenario, the morbidity, mortality, and case fatality rates were 35.53%, 5.32%, and 14.97%, respectively. In comparison with earlier research conducted in Egypt, the morbidity rate of LSD in this study was higher than percentages recorded in these studies [33, 34], with percentages of 31.2% and 28.9%, respectively. In contrast, the authors in [35] found that Egyptian cattle had a 100% higher LSD morbidity rate with lower mortality and a case fatality rate of 1.8% for both. The host immune status, the viability of mechanical vectors and the veterinary authority’s vaccination of the entire cow herd in the region all influence this difference in the morbidity rate [36].

In this study, age significantly influenced the prevalence of LSD, with animals aged 1–3 years being more susceptible to infection than those at younger or older ages. This triggers the result documented by Elhaig et al. [37] and Molla et al. [38], but it is dissimilar with Sarkar et al. [39], who recorded that calves under a year were the most susceptible. Placing the calves in a separate barn reduced their vulnerability to biting flies [40]. Additionally, the locality had a significant role in the outbreak of LSD; the Al‐Ismailia governorate has recorded a higher morbidity rate (42.5%) than Al‐Sharkia (30.49%) and Al‐Beheira (28.57%). Environmental variance, sample population differences, vector activity, and testing procedures, all are responsible for morbidity rate fluctuations [41]. Furthermore, there was a higher risk of LSDV infection in cattle kept on flock grazing or using communal water sources. As mixing animals from various herds could lead to greater interaction and virus transmission [42] that enhanced when biting flies from various sources feed on cattle repeatedly [30]. Consistent with the current results, applying fly repellent reduced the prevalence of LSD, as vector management is one of the most critical strategies for reducing the disease’s spread [43]. This scenario underscores the critical importance of implementing screening tests and quarantine observation intervals for newly introduced animals, given that these interventions could potentially increase the incidence of LSD. This aligned with result recorded by Selim et al. [44].

Early identification of the clinical signs of LSDV subsequent laboratory confirmation is critical for its control [45]. This investigation focused on isolating LSDV on CAM of SPF‐ECEs, revealing that 88% of skin nodule biopsies and 42.9% of nasal swabs displayed characteristic pock lesions matched well with the earlier studies [45, 46]. The comprehension of CaPVs pathogenesis indicates that skin lesions are optimal for viral isolation, remaining isolatable for about 35 days after nodules appear [47]. Subsequently, this study tested the isolation of LSDV on MDBK cell line achieving a success rate of 91.46% overall, with 94.67% from skin nodule biopsies and 57% from nasal swabs. The notable CPE included cellular rounding and cluster aggregation, corroborating with earlier research [46, 48]. The findings indicated greater specificity for LSDV isolation in MDBK compared to ECE, attributed to higher LSDV titers obtained 5‐6 days postinoculation [49].

In this study, conventional PCR effectively identified and confirmed LSDV, detecting viral DNA (958‐bp) in 95.65% of nasal swab samples and 100% of skin nodule biopsies. The PCR assay showed 100% sensitivity for skin biopsies, a result that corroborates the findings reported in [3, 50]. The high detection rate viral DNA in skin samples is attributed the specific viral tropism for cutaneous tissues and its maintained high concentrations, a concept reinforced by the work of Bowden et al. [51] who noted skin biopsies tested positive for LSDV for several months after infection. This aligns with prior studies showing that skin tissues, nasal swabs, and saliva are more effective for LSDV genome detection than blood samples [52], due to the brief viremia phase of LSDV (4–11 days postinfection) [53]. Overall, LSDV was detected in all 21 skin nodules (100%) in infected cattle, aligning with clinical diagnosis. However, low detection in nasal swabs was attributed to intermittent viral shedding from Day 11 to day 38 postinfection [54]. Live attenuated vaccines for LSD in cattle can sometimes lead to “Neethling disease,” characterized by skin nodules [55], appearing 8–18 days postvaccination [56]. To address adverse reactions, DIVA assays are necessary to distinguish between vaccine and wild‐type LSDV strains. Therefore, rapid and precise laboratory assays are essential for the verification of clinical diagnosis during LSD outbreak [57]. So, this study highlighted the role of the EEV glycoprotein gene as it is essential for CaPVs to infect cattle by enable the virus to attach and enter host cells [58] where LSDV126 gene region of the nonvirulent strain presented a 27 base pair discrepancy compared to the virulent virus [19].

In this scenario, the partial sequencing of EEV glycoprotein of all three virulent field isolates exhibited only one SNPs between the isolates of Al‐Sharkia, and in Al‐Ismailia and Al‐Menofia isolates outbreak, this aligned with Elsheikh et al. [34] and Saad et al. [59], while differed in two and/or three SNP with previous field isolates. This result is consistent with the well‐established fact that CaPVs exhibit significant levels of conservation within and between their species [60]. The BLAST analysis revealed a high sequence similarity of 99.85%–100% among field isolates and exhibited great homology (99.7%–100%) with Egyptian isolates, indicating no circulating variants among different cattle breeds in Egypt. This might be because LSDV has a high degree of genetic stability and just a few numbers of LSDV sequences are utilized, together with partial sequence of only one gene [52]. Also, these isolates showed highly nucleotide sequence homology 100%–99.85% with virulent strains from India, Austria, South Africa, Israel, Russia, Bangladesh, Kazakhstan, and China. This result aligned with Kara et al. [61] who claimed that the genomes of CaPVs are evolutionarily conserved, with about 95% similarity between the LSDV, SPPV, and GTPV. Additionally, the occurrence of the same LSDV outbreaks was due to livestock smuggling and open borders. In contrast, the detected genetic diversity from other contemporary isolates and LSDV vaccine might be as a result of genomic recombination when distinct LSDV strains spontaneously infect a host cell, leading to the exchange of genetic material between viral genomes [62] and the presence of recombinant LSDV further emphasizes the potential for genetic exchange and evolution within the LSDV population [63].

The phylogenetic analysis revealed that LSDV is closely related to other LSDV strain isolates while showing significant divergence from GTPV and SPPV, implying that sheep pox vaccination did not lead to these clinical manifestations. This agrees with findings by Saltykov et al. [64], who found no genetic material from the SPPV vaccine in analyzed samples, indicating no coinfection with SPPV in the cattle. This analysis faced limitations in achieving accurate genetic distinctions due to insufficient data and the use of partial genomic sequences rather than whole genome sequencing [65].

EEV glycoprotein gene sequence alignment exhibited distinct 27‐nucleotide insertion which is a characteristic of common LSD field isolates, similar to LSDVs from Bangladesh, LSD Neethling 2490, LSD BLG172, and LSDV Kenya that differentiating them from Neethling‐like vaccines. Conversely, the imported Neethling vaccine showed a deletion of this 27‐nucleotide as (ARRIAH LSD VAC). This dissimilar with Elsheikh et al. [34] who conducted that local vaccine that registered with accession number OL960035 had 27‐nucleotide. The observed variance may result from the analysis of a partial genomic sequence of the EEV glycoprotein gene, rendering local vaccinations unsuitable for DIVA. Future applications in Egypt could utilize advanced methods such as an ORF154‐based ELISA, where vaccinated animals do not produce antibodies against ORF3/ORF154 proteins, unlike those infected with a field strain of LSDV [66]. In addition, sequence analysis revealed significant variations in amino acid sequences between attenuated Neethling vaccinal strains and virulent LSDV field strains, which may impact virus functionality and pathogenesis. Variations in the EEV glycoprotein highlight the complex relationship between viral mutation and protein function [65].

Nowadays, field outbreaks of LSD associated with recombinant strains of the vaccine‐like LSDV have been documented in Russia [67], Kazakhstan [68], China [69], and Vietnam [70]. There is an opportunity that vaccine‐like recombinant LSDV will be introduced into Egypt due to the prevalence of various recombinant LSDVs within this geographical area (comprising Russia, Kazakhstan, China, and Vietnam); therefore, vigilant monitoring for the emergence of such recombinant strains within Egypt is very essential.

## 5. Conclusion

The outcomes of this study highlight significant insights that enhance the understanding of the epidemiology and molecular analysis of LSDV in four governorates in Egypt. The URL analysis revealed that age, locality, communal water source, and lack of quarantine, all have significant association with the outbreak of LSD. Nevertheless, these independent risk factors should be interpreted cautiously. The partial sequence of EEV gene was chosen to apply DIVA assay using ARRIAH LSD VAC and demonstrated that the field isolates have a distinct 27‐nucleotide insertion in contrast to ARRIAH LSD VAC. These data highlight the transboundary character of LSDV from a neighboring country by indicating the existence of genetically identical LSDVs. The results emphasize the necessity for continuous surveillance and monitoring of the disease to avert its further spread. Additionally, innovative strategic policies should be formulated to effectively manage and eradicate LSD, as well as public awareness campaigns are essential to augment vector control strategies and improve the comprehension of the disease. This study is limited to a partial sequence of a single gene of LSDV. Therefore, we recommend employing advanced molecular assays for whole genome sequencing to elucidate evolutionary history and monitor the adaptation‐driven genetic diversity within the LSDV genome in the field, as well as its impact on pathogenesis and epidemiology.

## Author Contributions

Heba Hassan El‐Nady, Mohamed Ibrahim Eissa, Naser Zeidan Abou‐Zeid, Elshaima Mohamed Fawzi, and Amal Mokhtar Abd El‐Raof participated in the concept development and study execution. While Yousry Abdelfatah El Shazly, Mohamed Mansour Bakrash, Ahmed Mansour, and Abdelrhman Awad Sobeih conducted data analysis and interpretation processes. The first draught of the manuscript was written by Heba Hassan El‐Nady. The manuscript revision and editing were done by Mohamed Ibrahim Eissa, Naser Zeidan Abou‐Zeid, and Elshaima Mohamed Fawzi. All of the study’s data were completely accessible to the authors, who also accepted responsibility for the accuracy of the data analysis and final submission.

## Funding

No funding was provided for this research.

## Ethics Statement

This study was designed after receiving approval from Zagazig University’s Animal Care and Use Committee (ZU‐IACUC/2/F/41/2025), and all processes were completed in compliance with the relevant regulations and rules. The study was conducted in accordance with ARRIVE criteria.

## Consent

The authors have nothing to report.

## Conflicts of Interest

The authors declare no conflicts of interest.

## Data Availability

The data that support the findings of this study are available from the corresponding author upon reasonable request.
